# Supplementary material for: Analysis of the effects of isolation piles on the basal stability of foundation pits against upheaval based on continuous velocity fields
Source: PLoS One. 2023 Apr 19;18(4):e0283695. doi: 10.1371/journal.pone.0283695 (PMC10115301; doi:10.1371/journal.pone.0283695)
Supplement: Attachment — Submitted filename: Response.pdf [file pone.0283695.s014.pdf]

The authors have derived a formula for the coefficient of basal stability against upheaval under the action of isolation piles by simplifying the pile-soil relationship and have systematically examined the impact of isolation pile parameters on basal stability against upheaval using the theory of continuous velocity fields and the upper bound limit analysis method.

I recommend the manuscript for publication and my suggestions are as follows:

1. Please explain briefly the method you have adopted in calculating the basal stability of foundation pits against upheaval considering the supporting capacity of isolation piles in last paragraph of “Section 1. Introduction”.
2. Please cite more references in support of your argument that isolation piles can effectively limit soil displacement. A suggestion is the following study
   “Mechanism of Isolating Piles in Reducing Tunnel Settlement of Hong Kong-Zhuhai-Macao Bridge Project”
3. Please comment if the authors have produced Table 1 themselves or have taken from some standard. If latter is true, please cite the source.
4. Please cite the source of Table 2.
5. Please correct **t** is description of Eq. (18).
6. Although the authors have not considered the effects of isolation piles on the form of velocity fields, but they should comment on the impact of this effect on the continuous velocity field considered.
7. Please cite reference of the method “shear strength reduction finite element method (SSRFEM)” mentioned in “Section 5. Numerical Simulation”.
8. The details of numerical model developed and numerical tool used are not provided in “Section 5. Numerical Simulation”.
9. Please comment on the discrepancies seen in the validation of numerical results with the theoretical results in Fig 2.
10. Please consult a native English speaker for proof reading the manuscript.
